# Correlation of the BACH1 Pro919Ser polymorphism with breast cancer risk: A literature-based meta-analysis and meta-regression analysis

**DOI:** 10.3892/etm.2013.1148

**Published:** 2013-06-07

**Authors:** JING SHI, JIANHUA TONG, SHUANG CAI, XIUJUAN QU, YUNPENG LIU

**Affiliations:** 1 Departments of Medical Oncology, The First Hospital of China Medical University, Shenyang, Liaoning 110001, P.R. China; 2 Drug Clinical Trials, The First Hospital of China Medical University, Shenyang, Liaoning 110001, P.R. China; 3 Pharmacy, The First Hospital of China Medical University, Shenyang, Liaoning 110001, P.R. China

**Keywords:** BACH1, polymorphism, breast cancer, meta-analysis, meta-regression analysis

## Abstract

Recent investigations have suggested that common genetic polymorphisms in BRCA1-associated C-terminal helicase 1 (BACH1) are important in the development of breast cancer. However, individually published studies and previous meta-analyses have demonstrated inconclusive results. The aim of this meta-analysis was to derive a more precise estimation of the correlation between a common polymorphism [proline (Pro) 919 serine (Ser); rs4986764 C>T] in the BACH1 gene and susceptibility to breast cancer. A literature search of PubMed, Embase, Web of Science and Chinese BioMedicine (CBM) databases was conducted on articles published prior to March 1, 2013. Crude odds ratios (ORs) with 95% confidence intervals (CIs) were calculated. Eleven case-control studies were included with a total of 6,903 breast cancer cases and 8,154 healthy controls. The meta-analysis results revealed that the BACH1 919Ser polymorphism may be correlated with a decreased risk of breast cancer among Caucasian populations (Ser allele versus Pro allele: OR=0.90, 95% CI=0.86–0.95; Pro/Ser + Ser/Ser versus Pro/Pro: OR=0.90, 95% CI=0.84–0.98; Ser/Ser versus Pro/Pro + Pro/Ser: OR=0.84, 95% CI=0.76–0.92; Ser/Ser versus Pro/Pro: OR=0.81, 95% CI=0.73–0.91; Ser/Ser versus Pro/Ser: OR=0.86, 95% CI=0.78–0.95), although not among Asian populations. Further subgroup analyses indicated that there were significant correlations between the BACH1 919Ser polymorphism and a decreased risk of breast cancer in postmenopausal females, females with a family history of breast cancer and females without BRCA1/2 mutations. Univariate and multivariate meta-regression analyses revealed that none of the factors explained the heterogeneity (all P>0.05). The present meta-analysis suggested that the BACH1 919Ser polymorphism may decrease the risk of breast cancer among Caucasian populations, particularly in postmenopausal females with a family history of breast cancer and without BRCA1/2 mutations.

## Introduction

Breast cancer is a major public health concern threatening the health of females worldwide and representing 4% of all female mortalities due to cancer (1). It is the most common type of cancer among females in developing and developed countries (2). The incidence and mortality rates of breast cancer have considerable global variations, with the highest rates observed in Europe and North America and the lowest in Asia (3). Consistent with other forms of cancer, breast cancer is a byproduct of multiple environment and hereditary risks (4). Futhermore, family history is an influential factor in the development of the disease. In a population-based study, mutations in the two predominant breast cancer susceptibility genes, BRCA1 and BRCA2, accounted for approximately 20% of familial breast cancer diagnoses (5). Studies have revealed that certain rare and low-frequency variants also have an impact on the risks of developing breast cancer, including TP53, PTEN, STK11, ATM, CHEK2 and BRCA1-interacting protein C-terminal helicase 1 (BACH1) genes (6,7).

BACH1, also known as FANCJ or BRIP1, interacts with the BRCA1 C-terminal (BRCT) repeats of BRCA1 and the formed complex contributes to the BRCA1-interrelated double-strand break repair function (8). The human BACH1 gene is located on chromosome 17q22, distal to the BRCA1 gene located at 17q21, a region that is frequently altered in breast cancer. The BACH1 gene spans 180 kbps, comprising 20 exons and encodes a protein that is 1,249 amino acids long (9). Based on its interactions with BRCA1, the BACH1 gene is considered a potential breast cancer susceptibility gene (10). The interrelation of the gene with cancer susceptibility was identified by the direct and functional interaction between BACH1 and BRCA1, known as a classic tumor suppressor (11). Previously, it was demonstrated that the interaction of the BRCTs with BACH1 depends on the phosphorylation of BACH1 at S990 (12). Numerous frequently-occurring mutations in the BACH1 gene, particularly the most common polymorphism, proline (Pro) 919 serine (Ser) (rs4986764 C>T), have been identified and have provided indications of the function of BACH1 in breast carcinogenesis (11).

Several studies have suggested that the BACH1 Pro919Ser polymorphism may be important in increasing susceptibility to breast cancer (11,13–16). By contrast, certain other studies have suggested that the BACH1 Pro919Ser polymorphism is not correlated with an increased risk of breast cancer (17–22). A recent meta-analysis of eight case-control studies by Pabalan *et al* evaluated the correlations of three functional polymorphisms (Pro919Ser, C47G and G64A) in the BACH1 gene with breast cancer risk (23). These findings indicated that a heterozygous genotype (Pro/Ser) of the BACH1 Pro919Ser polymorphism may be correlated with an increased susceptibility to breast cancer risk in premenopausal females under the heterozygous model. However, the study failed to observe increased risks of breast cancer under other genetic models. There were three main reasons for these negative results, including the fact that three case-control studies were not searched and included by the previous meta-analysis, which resulted in the analysis having a relatively small sample size. Furthermore, in the previous meta-analysis, the authors only performed subgroup analyses based on ethnicity and menopausal status in the exploration of the sources of heterogeneity. Numerous additional factors may also have resulted in the observed heterogeneity, such as differences in genotyping methods, countries and regions, the source of the cases and controls and the quality score of the included studies. Moreover, univariate and multivariate meta-regression analyses were not used in the previous meta-analysis to explore possible sources of heterogeneity among the studies. The aim of the present study was to update previous meta-analyses, as well as to provide a more comprehensive and reliable conclusion on the correlations between the BACH1 Pro919Ser polymorphism and breast cancer risk.

## Materials and methods

### Literature search

Relevant papers published prior to March 1, 2013 were identified through a search of PubMed, Embase, Web of Science and China BioMedicine (CBM) databases using the terms: (‘genetic polymorphism’ or ‘polymorphism’ or ‘SNP’ or ‘single nucleotide polymorphism’ or ‘gene mutation’ or ‘genetic variants’) and (‘breast neoplasms’ or ‘breast cancer’ or ‘breast tumor’ or ‘breast carcinoma’) and (‘BRCA1-interacting protein 1’ or ‘BRIP1 protein, human’ or ‘BACH1’ or ‘BRIP1’ or ‘BRAH1’ or ‘BRCA1 interacting protein C-terminal helicase 1’). The references from the eligible articles or textbooks were also reviewed in order to determine additional potential sources. Disagreements were resolved through discussions between the authors.

### Inclusion and exclusion criteria

Studies included in the present meta-analysis had to meet the following criteria: i) case-control studies had to focus on the correlation between the BACH1 Pro919Ser polymorphism and breast cancer risk; ii) any diagnoses of patients with cancer had to be confirmed by pathological examinations; iii) the published data on the frequencies of alleles or genotypes had to be sufficient. The exclusion criteria comprised case-control studies not focusing on the correlation between the BACH1 Pro919Ser polymorphism and breast cancer risk, duplicates of previous publications, studies based on incomplete data, and meta-analyses, letters, reviews and editorial articles.

### Data extraction

Data from the published studies were extracted independently by two authors into a standardized form. For each study, the following characteristics were assessed: The first author, year of publication, country, language, study design, ethnicity of subjects, number of subjects, gender ratio, mean age, type of cancer, detection sample, genotyping method, allele and genotype frequencies of single-nucleotide polymorphisms (SNPs) and evidence of the Hardy-Weinberg equilibrium (HWE) in controls. In cases of conflicting evaluations, disagreements were resolved through discussions between the authors.

### Quality assessment of included studies

Two authors independently assessed the quality of the included studies according to the modified Strengthening the Reporting of Observational Studies in Epidemiology (STROBE) quality score systems (24). Forty assessment items interrelated with the quality appraisal were used in the meta-analysis, with scores of 0–40. On the basis of the scores of the studies, the included studies were classified into three levels: Low quality (0–19), moderate quality (20–29) and high quality (30–40), respectively. Disagreements were resolved through discussions between the authors.

### Statistical analysis

Crude odds ratios (ORs) with 95% confidence intervals (CIs) were calculated under five genetic models: The allele (Ser versus Pro), dominant (Ser/Ser + Pro/Ser versus Pro/Pro), recessive (Ser/Ser versus Pro/Pro + Pro/Ser), homozygous (Ser/Ser versus Pro/Pro) and heterozygous (Ser/Ser versus Pro/Ser) models. The statistical significance of the pooled ORs was assessed using the Z-test. Interstudy variations and heterogeneities were estimated using Cochran’s Q-test, with P_h_<0.05 indicating a statistically significant heterogeneity (25). Furthermore, the effects of heterogeneity were quantified using the I^2^ test (range, 0–100%), which represented the proportion of interstudy variability that was able to be contributed to heterogeneity rather than to chance (26). When a significant Q-test with P_h_<0.05 or I^2^>50% indicated that heterogeneity existed among the studies, the random-effects model (DerSimonian-Laird method) was conducted for the meta-analysis; otherwise, the fixed-effects model (Mantel-Haenszel method) was used. To explore the sources of heterogeneity, a subgroup analysis was performed according to ethnicity, the source of the cases, genotyping method, menopausal status, family history and BRCA 1/2 mutations. In addition, univariate and multivariate regression analyses were conducted (27). Sensitivity analysis was performed through the omission of each study in turn to assess the quality and consistency of the results, while Begg’s funnel plots were used to detect publication biases. Egger’s linear regression test was also used to evaluate the publication biases (28). A χ^2^ test was used to test whether the genotype frequencies of the controls were in HWE. P-values were two-sided, and analyses were calculated using Stata software, version 12.0 (Stata Corp., College Station, TX, USA). P<0.05 was considered to indicate a statistically significant difference.

## Results

### Characteristics of included studies

**Figure 1.**
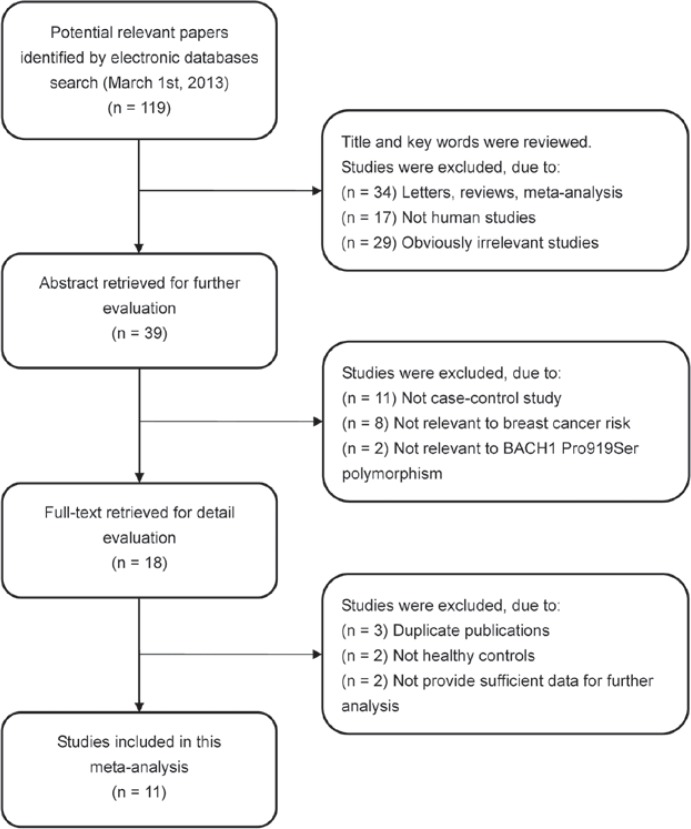
Flow chart of the study selection procedure. Eleven case-control studies were included in this meta-analysis. BACH1, BRCA1-associated C-terminal helicase 1.

In accordance with the inclusion criteria, 11 case-control studies (11,13–22) were included in the meta-analysis and 108 were excluded. The flow chart of the study selection process is shown in Fig. 1. The publication years of the included studies ranged from 2003 to 2011. A total of 15,057 subjects were involved in the meta-analysis, including 6,903 breast cancer cases and 8,154 healthy controls. All diagnoses of breast cancer were confirmed by pathological examinations. Six studies used hospital-based cases, two used population-based cases and the remaining three studies used family-based cases. The source of the healthy controls in all the included studies was from the general population (population-based). The DNA samples used for examination of the BACH1 Pro919Ser polymorphism were extracted from the blood in all the included studies. The genotyping methods included denaturing high-performance liquid chromatography (DHPLC), Microarray, TaqMan assay, MassArray, polymerase chain reaction-restriction fragment length polymorphism (PCR-RFLP) and PCR-single strand conformation polymorphism (PCR-SSCP). Eight of the studies were conducted in Caucasian populations and three in Asian populations. The HWE test was conducted on the genotype distribution of the controls in all 11 studies. None of the studies deviated from the HWE (all P>0.05). The quality scores of the 11 included studies were all >20 (moderate-high quality). The characteristics and methodological quality of the included studies are shown in Table I.

### Quantitative data synthesis

**Figure 2.**
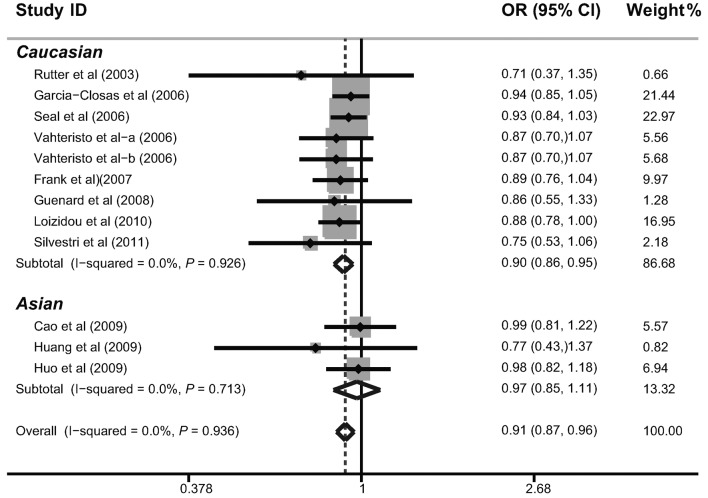
Subgroup analysis by ethnicity for the correlation between the BRCA1-associated C-terminal helicase 1 (BACH1) proline (Pro) 919 serine (Ser) polymorphism and breast cancer risk under the allele model. OR, odds ratios; 95% CI, 95% confidence interval.

A summary of the meta-analysis findings of the correlation between the BACH1 Pro919Ser polymorphism and breast cancer risk is provided in Table II. No heterogeneity was observed with any of the genetic models (all P_h_>0.05 and I^2^< 50%); therefore, the fixed effects model was used. The results of the meta-analysis revealed that the BACH1 919Ser polymorphism was correlated with a decreased risk of breast cancer (Ser allele versus Pro allele: OR=0.91, 95% CI=0.87–0.96, P<0.001; Pro/Ser + Ser/Ser versus Pro/Pro: OR=0.92, 95% CI=0.86–0.99, P=0.022; Ser/Ser versus Pro/Pro + Pro/Ser: OR=0.83, 95% CI=0.76–0.92, P<0.001; Ser/Ser versus Pro/Pro: OR=0.81, 95% CI=0.73–0.90, P<0.001; Ser/Ser versus Pro/Ser: OR=0.85, 95% CI=0.77–0.94, P=0.001). Further subgroup analysis by ethnicity indicated that the BACH1 919Ser polymorphism may decrease the risk of breast cancer among Caucasian populations (Ser allele versus Pro allele: OR=0.90, 95% CI=0.86–0.95, P<0.001; Pro/Ser + Ser/Ser versus Pro/Pro: OR=0.90, 95% CI=0.84–0.98, P=0.012; Ser/Ser versus Pro/Pro + Pro/Ser: OR=0.84, 95% CI=0.76–0.92, P<0.001; Ser/Ser versus Pro/Pro: OR=0.81, 95% CI=0.73–0.91, P<0.001; Ser/Ser versus Pro/Ser: OR=0.86, 95% CI=0.78–0.95, P=0.002). However, the results did not suggest a correlation among Asian populations (Fig. 2).

**Figure 3.**
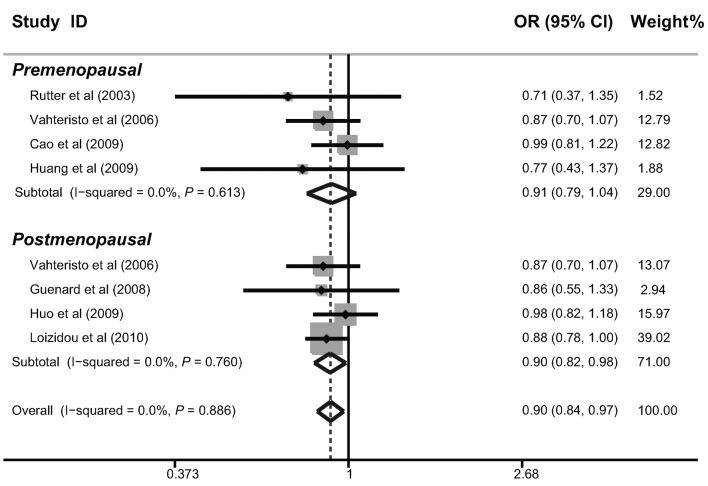
Subgroup analysis by menopausal status for the correlation between the BRCA1-associated C-terminal helicase 1 (BACH1) proline (Pro) 919 serine (Ser) polymorphism and breast cancer risk under the allele model. OR, odds ratios; 95% CI, 95% confidence interval.

**Figure 4.**
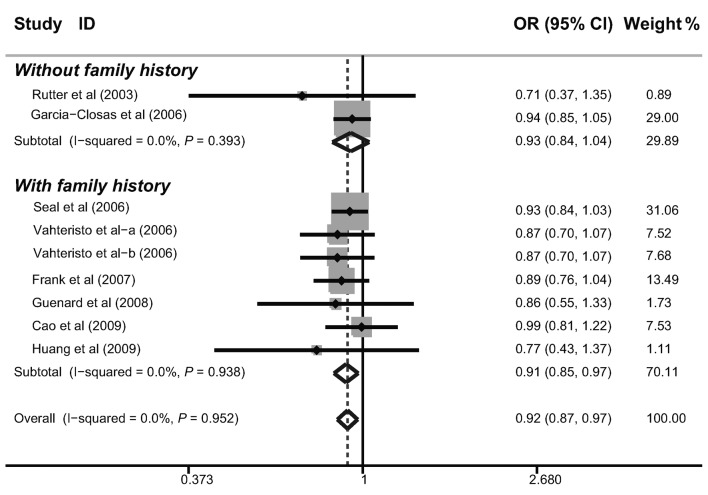
Subgroup analysis by family history of breast cancer for the correlation between the BRCA1-associated C-terminal helicase 1 (BACH1) proline (Pro) 919 serine (Ser) polymorphism and breast cancer risk under the allele model. OR, odds ratios; 95% CI, 95% confidence interval.

**Figure 5.**
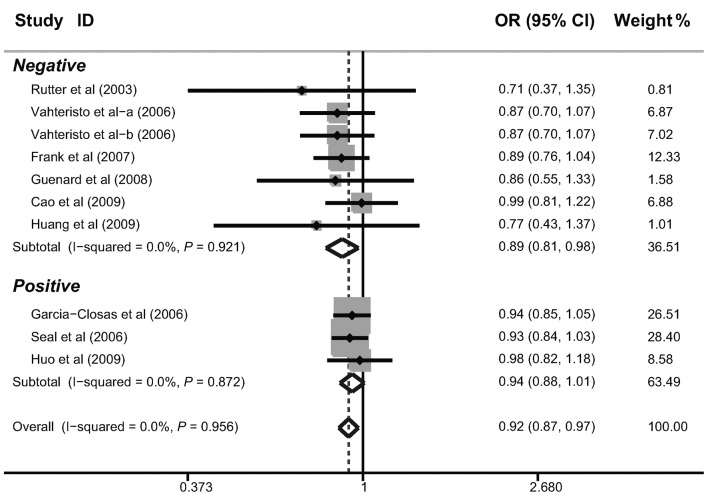
Subgroup analysis by BRCA1/2 mutations for the correlation between the BRCA1-associated C-terminal helicase 1 (BACH1) proline (Pro) 919 serine (Ser) polymorphism and breast cancer risk under the allele model. OR, odds ratios; 95% CI, 95% confidence interval.

In the investigation into factors that may have had a potential impact on the results, further subgroup analyses were performed according to the source of the cases, genotyping method, menopausal status, family history and BRCA1/2 mutations. The subgroup analysis by the source of the cases indicated that there were significant correlations between the BACH1 919Ser polymorphism and a decreased risk of breast cancer in hospital-based and family-based studies (as shown in Table II). Similar correlations were also observed in post-menopausal females, females with a family history of breast cancer and females without BRCA1/2 mutations (Figs. 3–5).

### Meta-regression and sensitivity analyses

**Figure 6.**
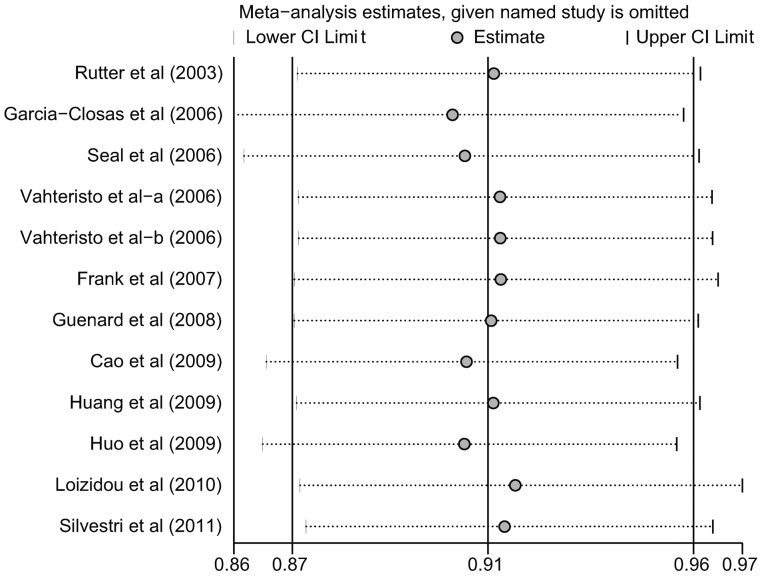
Sensitivity analysis of the correlation between the BRCA1-associated C-terminal helicase 1 (BACH1) proline (Pro) 919 serine (Ser) polymorphism and breast cancer risk under the allele model. OR, odds ratios; 95% CI, 95% confidence interval.

Univariate and multivariate meta-regression analyses were used to explore the possible sources of heterogeneity among the studies (Table III). The results revealed that none of the factors explained the heterogeneity (all P>0.05). Sensitivity analysis was performed to assess the effect of each individual study on the pooled ORs by the omission of individual studies. The analysis results suggested that no individual studies significantly affected the pooled OR of the correlation between the BACH1 919Ser polymorphism and breast cancer risk under the allele model (Fig. 6), indicating that the results of the analysis were statistically reliable.

### Publication bias evaluation

**Figure 7.**
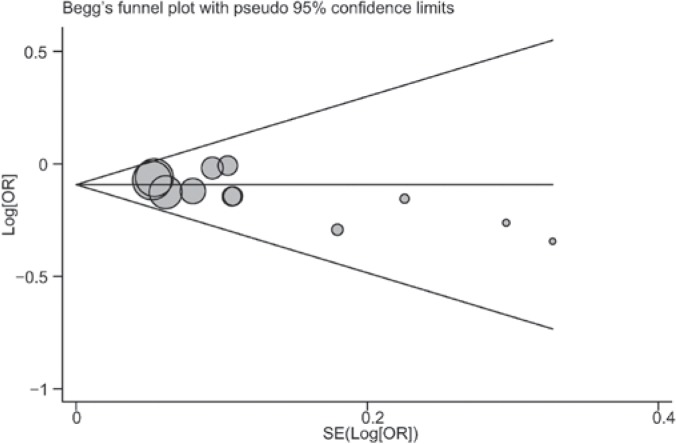
Begg’s funnel plot of the meta-analysis of the BRCA1-associated C-terminal helicase 1 (BACH1) proline (Pro) 919 serine (Ser)polymorphism and breast cancer risk under the allele model. Each point represents a separate study for the indicated correlation. Log[OR], natural logarithm of odds ratios (OR); SE, standard error; horizontal line, mean magnitude of the effect.

The publication biases within the available study results may not have been representative of all of the results from the study. Begg’s funnel plots and Egger’s linear regression tests were performed to assess the publication biases in the included studies. The shape of the funnel plot for the correlation between the BACH1 919Ser polymorphism and breast cancer risk did not indicate any marked asymmetry (Fig. 7). In addition, no notable suggestions of publication bias under the allele model were observed with Egger’s test (t=−1.03, P=0.327).

## Discussion

The protein encoded by the BACH1 gene has been demonstrated to be important in the double-strand break (DSB) repair pathway (29). It is also involved in the maintenance of DNA stability during transition through interactions with BRCA1 via the BRCT repeats domain (8). This process is required for the establishment of the G2 cell-cycle checkpoint response to DNA damage in the progression of the cell cycle (12). The abnormal expression of BACH1 has been identified to be correlated with the risk of breast cancer due to its inability to mediate DNA recombination repair (30). Furthermore, monoallelic mutations in the BACH1 gene have been demonstrated to be the predominant factor leading to the overexpression of BACH1, and these mutations may increase the hereditary breast cancer susceptibility (10). Therefore, it was suggested that the BACH1 gene polymorphisms were functional and were correlated with breast cancer risk. At present, a total of eight BACH1 truncating mutations have been identified worldwide, and the Pro919Ser polymorphism, which codes for amino acid 919 of the BACH1 protein, has been demonstrated to be closely correlated with breast cancer susceptibility (7,17). Certain previous case-control studies and a recent meta-analysis have suggested that the BACH1 Pro919Ser polymorphism may be important in the development of breast cancer. However, the results from other investigations indicated that this polymorphism did not affect the susceptibility of an individual to breast cancer. There may be several reasons for this controversy, such as the differences in the study designs, sample sizes, the ethnicity of the subjects, the source of the cases and controls, genotyping methods and menopausal status (31). Therefore, the present meta-analysis was performed to provide a more comprehensive and reliable conclusion with regard to the correlation between the BACH1 Pro919Ser polymorphism and susceptibility to breast cancer.

In this meta-analysis, 11 case-control studies were included with a total of 6,903 breast cancer cases and 8,154 healthy controls. When all the eligible studies were pooled into the meta-analysis, the results indicated that the BACH1 919Ser polymorphism decreased the risk of breast cancer among Caucasian populations, although a similar correlation was not observed among Asian populations. While the precise functions and effects of the BACH1 genetic polymorphisms on an individual’s susceptibility to breast cancer among different populations have not yet been elucidated, a potential explanation is that inherited mutations in BACH1 may be interrelated with the changes in expression and function of DNA repair, thereby accounting for the interindividual differences in susceptibility to breast cancer (11). Further subgroup analyses revealed that there were significant correlations between the BACH1 919Ser polymorphism and a decreased risk of breast cancer in hospital-based and family-based studies. Similar correlations were also observed in postmenopausal females, females with a family history of breast cancer and females without BRCA1/2 mutations. By contrast with the previous meta-analysis, which indicated that the Pro/Ser genotype increased the risk of breast cancer in premenopausal females, the present analysis revealed a significant correlation between the BACH1 919Ser polymorphism and a decreased risk of breast cancer in postmenopausal females (23). Furthermore, the results of the present meta-analysis suggested that the BACH1 919Ser polymorphism may be correlated with a decreased risk of breast cancer in females with a family history of breast cancer and without BRCA1/2 mutations.

Consistent with previous meta-analyses (23), the present study demonstrated certain limitations, such as the fact that only 14 investigations were included. Therefore, the sample size was relatively small and may not have provided sufficient statistical power. Thus, additional studies with larger sample sizes are required to provide an accurate and more representative statistical analysis. Furthermore, as a type of a retrospective study, a meta-analysis may encounter recall or selection bias, and this may have potentially influenced the reliability of the results in the present study (32,33). Moreover, the lack of access to the original data from the studies limited the present meta-analysis with regard to evaluation of potential interactions between additional factors and breast cancer risks, such as gene-environment and gene-gene interactions (34).

In conclusion, the present meta-analysis indicated that the BACH1 919Ser polymorphism may decrease the risk of breast cancer among Caucasian populations, particularly in postmenopausal females with a family history of breast cancer and without BRCA1/2 mutations. These correlations have the potential to suggest a functional profiling of the involvement of the BACH1 gene in the development of breast cancer. In addition, the results may provide a foundation for additional studies in the diagnosis and clinical therapy of breast cancer. In consideration of the previously mentioned limitations of this analysis, detailed studies are required to confirm the results described. Studies investigating the effect of gene-environment interactions on breast cancer should also be conducted.

## Figures and Tables

**Table I.** Characteristics and methodological quality of the included studies in the meta-analysis.

First author	Year	Country	Ethnicity	Number		Source		Genotyping method	SNP ID	HWE test (P-value)	STROBE score
				Case	Control	Case	Control				
Rutter *et al*	2003	USA	Caucasian	58	30	HB	PB	DHPLC	rs4986764 (C>T)	0.876	25/40
García-Closas *et al*	2006	USA	Caucasian	1,898	1,514	PB	PB	Microarray	rs4986764 (C>T)	0.267	29/40
Seal *et al*	2006	UK	Caucasian	1,212	2,081	FB	PB	Microarray	rs4986764 (C>T)	0.340	27/40
Vahteristo *et al*	2006	Finland	Caucasian	888	736	HB	PB	TaqMan	rs4986764 (C>T)	0.318	30/40
Frank *et al*	2007	Germany	Caucasian	571	712	FB	PB	DHPLC	rs4986764 (C>T)	0.366	31/40
Guénard *et al*	2008	Canada	Caucasian	96	70	FB	PB	Microarray	rs4986764 (C>T)	0.690	28/40
Cao *et al*	2009	China	Asian	357	864	HB	HB	DHPLC	rs4986764 (C>T)	0.498	24/40
Huang *et al*	2009	China	Asian	50	150	HB	PB	DHPLC	rs4986764 (C>T)	0.866	32/40
Huo *et al*	2009	China	Asian	568	624	HB	PB	PCR-RFLP	rs4986764 (C>T)	0.359	26/40
Loizidou *et al*	2010	Cyprus	Caucasian	1,108	1,170	HB	PB	MassArray	rs4986764 (C>T)	0.242	32/40
Silvestri *et al*	2011	Italy	Caucasian	97	203	PB	PB	PCR-SSCP	rs4986764 (C>T)	0.850	33/40

PB, population-based; HB, hospital-based; FB, family-based; SNP, single-nucleotide polymorphism; DHPLC, denaturing high-performance liquid chromatography; PCR, polymerase chain reaction; RFLP, restriction fragment length polymorphism; SSCP, single strand conformation polymorphism; HWE, Hardy-Weinberg equilibrium; STROBE, Strengthening the Reporting of Observational Studies in Epidemiology.

**Table II.** Meta-analysis of the correlation between the BACH1 Pro919Ser polymorphism and breast cancer risk.

A. Allele model: Ser allele vs. Pro allele.				
Subgroup	OR	95% CI	P-value	P_h_-value
Overall	0.91	0.87–0.96	<0.001	0.936
Ethnicity				
Caucasian (n=8)	0.90	0.86–0.95	<0.001	0.926
Asian (n=3)	0.97	0.85–1.11	0.699	0.713
Source of cases				
Population-based (n=2)	0.92	0.83–1.02	0.122	0.216
Hospital-based (n=6)	0.90	0.84–0.98	0.010	0.817
Family-based (n=3)	0.91	0.84–0.99	0.037	0.848
Genotyping method				
DHPLC (n=4)	0.91	0.81–1.03	0.118	0.635
Microarray (n=3)	0.93	0.87–1.00	0.061	0.916
Others (n=4)	0.89	0.82–0.96	0.004	0.694
Menopausal status				
Premenopausal (n=4)	0.91	0.79–1.04	0.174	0.613
Postmenopausal (n=4)	0.90	0.82–0.99	0.021	0.760
Family history of breast cancer				
Yes (n=6)	0.91	0.85–0.97	0.007	0.938
No (n=2)	0.93	0.84–1.04	0.202	0.393
BRCA1/2 mutations				
Positive (n=3)	0.94	0.88–1.01	0.084	0.872
Negative (n=6)	0.89	0.81–0.98	0.013	0.921

B. Dominant model: Pro/Ser + Ser/Ser vs. Pro/Pro.				
Subgroup	OR	95% CI	P-value	P_h_-value
Overall	0.92	0.86–0.99	0.022	0.987
Ethnicity				
Caucasian (n=8)	0.90	0.84–0.98	0.012	0.999
Asian (n=3)	1.00	0.85–1.17	0.961	0.535
Source of cases				
Population-based (n=2)	0.93	0.80–1.08	0.327	0.871
Hospital-based (n=6)	0.93	0.83–1.03	0.157	0.806
Family-based (n=3)	0.91	0.80–1.03	0.126	0.945
Genotyping method				
DHPLC (n=4)	0.90	0.77–1.07	0.228	0.658
Microarray (n=3)	0.93	0.83–1.03	0.150	0.992
Others (n=4)	0.92	0.82–1.04	0.184	0.817
Menopausal status				
Premenopausal (n=4)	0.91	0.75–1.11	0.347	0.662
Postmenopausal (n=4)	0.93	0.82–1.06	0.270	0.703
Family history of breast cancer				
Yes (n=6)	0.91	0.83–1.01	0.079	0.975
No (n=2)	0.92	0.79–1.08	0.305	0.470
BRCA1/2 mutations				
Positive (n=3)	0.95	0.86–1.04	0.216	0.642
Negative (n=6)	0.90	0.79–1.03	0.138	0.947

C. Recessive model: Ser/Ser vs. Pro/Pro + Pro/Ser.				
Subgroup	OR	95% CI	P-value	P_h_-value
Overall	0.83	0.76–0.92	<0.001	0.779
Ethnicity				
Caucasian (n=8)	0.84	0.76–0.92	<0.001	0.712
Asian (n=3)	0.76	0.48–1.20	0.237	0.405
Source of cases				
Population-based (n=2)	0.87	0.73–1.04	0.116	0.074
Hospital-based (n=6)	0.78	0.67–0.91	0.002	0.998
Family-based (n=3)	0.86	0.74–1.01	0.059	0.790
Genotyping method				
DHPLC (n=4)	0.80	0.61–1.06	0.122	0.802
Microarray (n=3)	0.90	0.79–1.02	0.110	0.878
Others (n=4)	0.76	0.66–1.09	0.322	0.628
Menopausal status				
Premenopausal (n=4)	0.79	0.58–1.08	0.141	0.817
Postmenopausal (n=4)	0.78	0.66–0.92	0.004	0.996
Family history of breast cancer				
Yes (n=6)	0.84	0.74–0.95	0.006	0.909
No (n=2)	0.91	0.76–1.09	0.314	0.653
BRCA1/2 mutations				
Positive (n=3)	0.89	0.79–1.01	0.081	0.754
Negative (n=6)	0.79	0.67–0.93	0.006	0.998

D. Homozygous model: Ser/Ser vs. Pro/Pro.				
Subgroup	OR	95% CI	P-value	P_h_-value
Overall	0.81	0.73–0.90	<0.001	0.920
Ethnicity				
Caucasian (n=8)	0.81	0.73–0.91	<0.001	0.871
Asian (n=3)	0.79	0.49–1.26	0.317	0.292
Source of cases				
Population-based (n=2)	0.85	0.69–1.04	0.104	0.112
Hospital-based (n=6)	0.77	0.64–0.92	0.003	0.994
Family-based (n=3)	0.84	0.70–0.99	0.039	0.812
Genotyping method				
DHPLC (n=4)	0.75	0.55–1.04	0.083	0.660
Microarray (n=3)	0.87	0.76–1.01	0.070	0.949
Others (n=4)	0.75	0.63–0.89	0.001	0.810
Menopausal status				
Premenopausal (n=4)	0.74	0.49–1.12	0.155	0.671
Postmenopausal (n=4)	0.77	0.64–0.94	0.008	0.999
Family history of breast cancer				
Yes (n=6)	0.82	0.71–0.95	0.009	0.962
No (n=2)	0.88	0.71–1.08	0.223	0.512
BRCA1/2 mutations				
Positive (n=3)	0.87	0.75–1.00	0.051	0.899
Negative (n=6)	0.77	0.62–0.85	0.013	0.994

E. Heterozygous model: Ser/Ser vs. Pro/Ser.				
Subgroup	OR	95% CI	P-value	P_h_-value
Overall	0.85	0.77–0.94	0.001	0.722
Ethnicity				
Caucasian (n=8)	0.86	0.78–0.95	0.002	0.684
Asian (n=3)	0.72	0.44–1.16	0.177	0.618
Source of cases				
Population-based (n=2)	0.88	0.73–1.07	0.191	0.093
Hospital-based (n=6)	0.80	0.68–0.93	0.005	0.985
Family-based (n=3)	0.86	0.75–1.04	0.142	0.797
Genotyping method				
DHPLC (n=4)	0.83	0.62–1.12	0.222	0.963
Microarray (n=3)	0.92	0.80–1.06	0.241	0.867
Others (n=4)	0.77	0.66–0.90	0.001	0.532
Menopausal status				
Premenopausal (n=4)	0.81	0.58–1.13	0.217	0.993
Postmenopausal (n=4)	0.79	0.66–0.94	0.009	0.950
Family history of breast cancer				
Yes (n=6)	0.85	0.75–0.98	0.020	0.877
No (n=2)	0.93	0.77–1.13	0.480	0.829
BRCA1/2 mutations				
Positive (n=3)	0.91	0.79–1.04	0.166	0.600
Negative (n=6)	0.80	0.67–0.96	0.015	0.996

BACH1, BRCA1-associated C-terminal helicase 1; Ser, serine; Pro, proline; DHPLC, denaturing high performance liquid chromatography; OR, odds ratios; 95% CI, 95% confidence interval; P_h_-value, P-value of heterogeneity test.

**Table III.** Univariate and multivariate meta-regression analyses of potential sources of heterogeneity.

Heterogeneity factor	Analysis type	Coefficient	SE	z-value	P-value	95% CI	
						UL	LL
Publication year	Univariate	−0.005	0.015	−0.34	0.736	−0.034	0.024
	Multivariate	0.032	0.063	0.51	0.613	−0.092	0.155
Ethnicity	Univariate	0.077	0.073	1.05	0.293	−0.066	0.220
	Multivariate	−0.059	0.256	−0.23	0.817	−0.562	0.443
Source of cases	Univariate	−0.004	0.033	−0.12	0.906	−0.069	0.061
	Multivariate	0.001	0.049	0.02	0.984	−0.095	0.097
Genotyping method	Univariate	−0.019	0.036	−0.52	0.603	−0.088	0.051
	Multivariate	−0.024	0.065	−0.37	0.713	−0.150	0.103
Menopausal status	Univariate	0.010	0.036	0.28	0.777	−0.059	0.080
	Multivariate	−0.040	0.105	−0.38	0.704	−0.246	0.166
Family history of breast cancer	Univariate	−0.004	0.030	−0.13	0.893	−0.063	0.054
	Multivariate	0.004	0.078	0.05	0.957	−0.148	0.156
BRCA1/2 mutations	Univariate	−0.044	0.033	−1.34	0.179	−0.108	0.020
	Multivariate	−0.112	0.139	−0.81	0.419	−0.384	0.160

SE, standard error; 95% CI, 95% confidence interval; UL, upper limit; LL, lower limit.

## References

[1] Benson JR, Jatoi I (2012). The global breast cancer burden. Future Oncol.

[2] Ferlay J, Shin HR, Bray F, Forman D, Mathers C, Parkin DM (2010). Estimates of worldwide burden of cancer in 2008: GLOBOCAN 2008. Int J Cancer.

[3] Jemal A, Bray F, Center MM, Ferlay J, Ward E, Forman D (2011). Global cancer statistics. CA Cancer J Clin.

[4] Wernberg JA, Yap J, Murekeyisoni C, Mashtare T, Wilding GE, Kulkarni SA (2009). Multiple primary tumors in men with breast cancer diagnoses: a SEER database review. J Surg Oncol.

[5] (2000). Prevalence and penetrance of BRCA1 and BRCA2 mutations in a population-based series of breast cancer cases. Anglian Breast Cancer Study Group. Br J Cancer.

[6] Pharoah PD, Tyrer J, Dunning AM, Easton DF, Ponder BA, SEARCH Investigators (2007). Association between common variation in 120 candidate genes and breast cancer risk. PLoS Genet.

[7] Kuusisto KM, Bebel A, Vihinen M, Schleutker J, Sallinen SL (2011). Screening for BRCA1, BRCA2, CHEK2, PALB2, BRIP1, RAD50, and CDH1 mutations in high-risk Finnish BRCA1/2-founder mutation-negative breast and/or ovarian cancer individuals. Breast Cancer Res.

[8] Cantor SB, Bell DW, Ganesan S (2001). BACH1, a novel helicase-like protein, interacts directly with BRCA1 and contributes to its DNA repair function. Cell.

[9] Song H, Ramus SJ, Kjaer SK (2007). Tagging single nucleotide polymorphisms in the BRIP1 gene and susceptibility to breast and ovarian cancer. PLoS One.

[10] Cantor SB, Guillemette S (2011). Hereditary breast cancer and the BRCA1-associated FANCJ/BACH1/BRIP1. Future Oncol.

[11] Vahteristo P, Yliannala K, Tamminen A, Eerola H, Blomqvist C, Nevanlinna H (2006). BACH1 Ser919Pro variant and breast cancer risk. BMC Cancer.

[12] Yu X, Chini CC, He M, Mer G, Chen J (2003). The BRCT domain is a phospho-protein binding domain. Science.

[13] Huo X, Lu C, Huang X (2009). Polymorphisms in BRCA1, BRCA1-interacting genes and susceptibility of breast cancer in Chinese women. J Cancer Res Clin Oncol.

[14] Huang J, Tang LL, Hu Z (2008). BRCA1 and BRCA2 gene mutations of familial breast cancer and early-onset breast cancer from Hunan Province in China. China Oncology.

[15] Cao AY, Huang J, Hu Z (2009). Mutation analysis of BRIP1/BACH1 in BRCA1/BRCA2 negative Chinese women with early onset breast cancer or affected relatives. Breast Cancer Res Treat.

[16] Seal S, Thompson D, Renwick A, Breast Cancer Susceptibility Collaboration (UK) (2006). Truncating mutations in the Fanconi anemia J gene BRIP1 are low-penetrance breast cancer susceptibility alleles. Nat Genet.

[17] Silvestri V, Rizzolo P, Falchetti M (2011). Mutation analysis of BRIP1 in male breast cancer cases: a population-based study in Central Italy. Breast Cancer Res Treat.

[18] Loizidou MA, Cariolou MA, Neuhausen SL (2010). Genetic variation in genes interacting with BRCA1/2 and risk of breast cancer in the Cypriot population. Breast Cancer Res Treat.

[19] Guénard F, Labrie Y, Ouellette G, Joly Beauparlant C, Simard J, Durocher F, INHERIT BRCAs (2008). Mutational analysis of the breast cancer susceptibility gene BRIP1 /BACH1/FANCJ in high-risk non-BRCA1/BRCA2 breast cancer families. J Hum Genet.

[20] Frank B, Hemminki K, Meindl A (2007). BRIP1 (BACH1) variants and familial breast cancer risk: a case-control study. BMC Cancer.

[21] García-Closas M, Egan KM, Newcomb PA (2006). Polymorphisms in DNA double-strand break repair genes and risk of breast cancer: two population-based studies in USA and Poland, and meta-analyses. Hum Genet.

[22] Rutter JL, Smith AM, Dávila MR (2003). Mutational analysis of the BRCA1-interacting genes ZNF350/ZBRK1 and BRIP1/BACH1 among BRCA1 and BRCA2-negative probands from breast-ovarian cancer families and among early-onset breast cancer cases and reference individuals. Hum Mutat.

[23] Pabalan N, Jarjanazi H, Ozcelik H (2013). Association between BRIP1 (BACH1) polymorphisms and breast cancer risk: a meta-analysis. Breast Cancer Res Treat.

[24] Gallo V, Egger M, McCormack V (2011). Strengthening the reporting of observational studies in epidemiology - molecular epidemiology (STROBE-ME): an extension of the STROBE Statement. PLoS Med.

[25] Higgins JP, Thompson SG (2002). Quantifying heterogeneity in a meta-analysis. Stat Med.

[26] Zintzaras E, Ioannidis JP (2005). Heterogeneity testing in meta-analysis of genome searches. Genet Epidemiol.

[27] Ioannidis JP, Patsopoulos NA, Rothstein HR (2008). Reasons or excuses for avoiding meta-analysis in forest plots. BMJ.

[28] Peters JL, Sutton AJ, Jones DR, Abrams KR, Rushton L (2006). Comparison of two methods to detect publication bias in meta-analysis. JAMA.

[29] Wong MW, Nordfors C, Mossman D (2011). BRIP1, PALB2, and RAD51C mutation analysis reveals their relative importance as genetic susceptibility factors for breast cancer. Breast Cancer Res Treat.

[30] Cantor S, Drapkin R, Zhang F (2004). The BRCA1-associated protein BACH1 is a DNA helicase targeted by clinically relevant inactivating mutations. Proc Natl Acad Sci USA.

[31] Rosenthal R, DiMatteo MR (2001). Meta-analysis: recent developments in quantitative methods for literature reviews. Annu Rev Psychol.

[32] Jüni P, Egger M (2009). PRISMAtic reporting of systematic reviews and meta-analyses. Lancet.

[33] Ioannidis JP, Lau J (1999). Pooling research results: benefits and limitations of meta-analysis. Jt Comm J Qual Improv.

[34] Dennis J, Hawken S, Krewski D (2011). Bias in the case-only design applied to studies of gene-environment and gene-gene interaction: a systematic review and meta-analysis. Int J Epidemiol.

